# The effect of non-compliance with diet and liquid restriction on fatigue in dialysis patients

**DOI:** 10.1590/1980-220X-REEUSP-2023-0251en

**Published:** 2024-05-24

**Authors:** Ridvan Bayram, Serkan Budak, Hicran YIldIz

**Affiliations:** 1 Bursa Uludag University, Faculty of Health Sciences, Department of Medical Nursing, Bursa, Turkey.; 2 Kütahya Health Sciences University, Simav Vocational School of Health Services, Department of Medical Services and Techniques, Kütahya, Turkey

**Keywords:** Fatigue, Diet, Dialysis, Therapeutics, Diet Therapy, Fadiga, Dieta, Diálise, Terapêutica, Dietoterapia, Fatiga, Dieta, Diálisis, Terapéutica, Dietoterapia

## Abstract

**Objective:**

This study was conducted to determine the relationship between non-compliance with dietary and fluid restriction, body mass index, and the severity of fatigue in dialysis patients.

**Method:**

A descriptive and cross-sectional study was conducted on 42 dialysis patients. The data were collected employing a “General Information Form”, Body Mass Index, “Fatigue Severity Scale”, and “Dialysis Diet and Fluid Non-Adherence Questionnaire.”. Data were evaluated using percentages, averages, one-way ANOVA, T-tests, and Pearson correlation tests.

**Results:**

The average number of days when the patients did not comply with their diet was 3.69 ± 4.85, and the average number of days when they did not comply with fluid restriction was 2.71 ± 5.02. The age and marital status of the patients were found to affect the fatigue severity. It was found that the cases were associated with creatinine and calcium values and the number of days they did not comply with fluid restriction.

**Conclusion:**

It was determined that there was no significant relationship between non-compliance with diet and fluid restriction and the severity of fatigue. It was found that the severity of fatigue was lower in patients who complied with diet and fluid restriction, although not significantly lower than in those who did not comply.

## RESUMO

**Objetivo:**

Este estudo foi conduzido para determinar a relação entre a não aderência à restrição alimentar e hídrica, o índice de massa corporal e a severidade da fadiga em pacientes recebendo diálise.

**Método:**

Foi realizado um estudo descritivo e transversal com 42 pacientes recebendo diálise. Os dados foram coletados empregando o “Formulário de informações gerais”, Índice de Massa Corporal, “Escala de Severidade da Fadiga” e “Dieta da Diálise e Escala de Incompatibilidade Hídrica”. Os dados foram avaliados usando porcentagens, médias, ANOVA unidirecional, testes T e testes de correlação de Pearson.

**Resultados:**

O número médio de dias que os pacientes não cumpriram os requisitos da dieta foi de 3,69 ± 4,85 e o número médio de dias que não cumpriram os requisitos da restrição hídrica foi de 2,71 ± 5,02. Foi constatado que a idade e o estado civil dos pacientes afetavam a severidade da fadiga. Foi constatado que os casos estavam associados aos valores de creatinina e cálcio e ao número de dias que não cumpriam os requisitos da restrição hídrica.

**Conclusão:**

Foi determinado que não houve relação significativa entre o não cumprimento dos requisitos da dieta e da restrição hídrica e a severidade da fadiga. Foi constatado que a severidade da fadiga foi menor nos pacientes que cumpriram os requisitos da dieta e restrição hídrica, embora não significativamente menor em comparação àqueles que não cumpriram.

## RESUMEN

**Objetivo:**

Este estudio se realizó para determinar la relación entre el incumplimiento de la restricción dietética y de líquidos, el índice de masa corporal y la gravedad de la fatiga en pacientes en diálisis.

**Método:**

Se realizó un estudio descriptivo y transversal sobre 42 pacientes en diálisis. Los datos fueron recolectados mediante el “Formulario de información general”, Índice de Masa Corporal, “Escala de gravedad de la fatiga” y “Escala de incompatibilidad de líquidos y dieta de diálisis”. Los datos se evaluaron utilizando porcentajes, promedios, unidireccional ANOVA, Pruebas T y pruebas de correlación de Pearson.

**Resultados:**

El promedio de días que los pacientes no cumplieron con su dieta fue de 3,69 ± 4,85, y el promedio de días que no cumplieron con la restricción de líquidos fue de 2,71 ± 5,02. Se descubrió que la edad y el estado civil de los pacientes afectan la gravedad de la fatiga. Se encontró que los casos estuvieron asociados con los valores de creatinina y calcio y el número de días que no cumplieron con la restricción de líquidos.

**Conclusión:**

Se determinó que no existía una relación significativa entre el incumplimiento de la dieta y la restricción de líquidos y la gravedad de la fatiga. Se encontró que la gravedad de la fatiga era menor en los pacientes que cumplían con la dieta y la restricción de líquidos, aunque no significativamente menor que en aquellos que no la cumplían.

## INTRODUCTION

Chronic kidney disease is defined in the KDIGO (Kidney Disease Improving Global Outcomes) 2012 guideline as a glomerular filtration rate (GFR) of less than 60 mL/min/1.73 m^2^ for more than three months or functional or structural impairment of the kidney for more than three months, and blood, urine analysis and/or radiological damage^(1)^. Chronic kidney disease is an incurable, progressive disease with high morbidity and mortality that is common in the general adult population, especially in people with diabetes mellitus and hypertension. As a result of this disease, many complications can be seen. To prevent complications that may occur in patients with chronic kidney disease, patients should periodically receive dialysis and medication, follow treatment and nutrition recommendations, and pay attention to fluid restrictions. If patients pay attention to these issues, hospitalisation periods are shortened, and morbidity and mortality rates are reduced. In addition, the patient’s compliance with treatment, medication, nutrition and fluid restriction affects the success rate of dialysis treatment, prognosis and subsequent quality of life^(1,2)^.

In patients with chronic kidney disease, nutritional status is significant. The primary purpose of nutritional therapy in these patients is to ensure that most of the daily amount of energy is met with fats and carbohydrates and consists of animal proteins, which have a biologically higher protein value in the body^(2,3)^. Protein restriction is recommended in the diets of these patients to prevent the progression of kidney disease. In these patient groups, factors such as insufficient oral intake, losses, surgical and medical diseases, increase in protein catabolism, and problems encountered in the endocrine and metabolic system play a role in dietary issues. The most common reason is insufficient food intake^(2)^. The studies have found that most patients receiving hemodialysis treatments receive inadequate calories and protein. It was thought that this was due to anorexia, nausea and vomiting, gastritis, oesophagitis, infection, oral and dental problems, anaemia and depression associated with increased urea levels. Nutritional issues are closely related to mortality and morbidity in patients with a diagnosis of chronic kidney disease and receiving hemodialysis^(1–3)^.

Nutritional status is significant in patients with chronic kidney disease. The main aim of nutritional therapy in these patients is to ensure that most of the needed daily energy amount is met from fats, carbohydrates and animal proteins with a higher biological protein value in the body^(2,3)^. Protein restriction is recommended in the diets of these patients to prevent the progression of renal disease. Factors such as inadequate oral intake, losses, surgical and medical diseases, increased protein catabolism, and problems encountered in the endocrine and metabolic system play a role in the emergence of dietary issues in these patient groups. The most common cause is inadequate food intake^(2)^. In studies conducted on the subject, it has been found that most patients receiving haemodialysis treatment have inadequate calorie and protein intake. It was thought to be caused by anorexia, nausea and vomiting, gastritis, oesophagitis, infection, oral and dental problems, anaemia and depression associated with an increase in urea. Nutritional issues are closely related to mortality and morbidity in patients with chronic kidney disease who are on haemodialysis^(1–3)^.

Fatigue is one of the most common symptoms in dialysis patients^(3,4)^. It is a condition that occurs due to an inflammatory process expressed by an increase in the amount of waste products, muscle weakness and fatigue. Fatigue in dialysis patients may be encountered due to inadequate production of erythropoietin hormone in the kidneys and decreased life span of erythrocytes due to anaemia, fluid-electrolyte imbalance and also muscle atrophy due to myopathy and neuropathy developing as a result of increased urea amount. Changes in the structures of energy and energy-forming enzymes, inability to remove metabolic waste products resulting from metabolic events from the body, sleep problems, psychological, social and environmental problems, and symptoms related to other existing diseases and treatment methods cause fatigue in dialysis patients^(5,6)^. Fatigue negatively affects how haemodialysis patients spend their leisure time, their relationships with family and friends, their quality of life and their eating habits^(4,7)^. Therefore, fatigue evaluation in dialysis patients and interventions to reduce fatigue should be planned. One of these interventions is to adapt the patient to fluid and dietary restrictions. In studies conducted with various patient groups to date, it has been found that there is a relationship between fatigue and nutrition^(2,3)^.

Studies focusing on dialysis patients have so far predominantly focused on the volume of fluid consumed, while research on adherence to fluid restriction guidelines in this demographic group has been markedly limited. However, there is no study in the literature in which diet and fluid restriction compliance were evaluated together. This study was conducted to determine the relationship between non-compliance with diet and fluid restriction and fatigue severity in dialysis patients.

## METHOD

### Design of Study

Descriptive and cross-sectional study.

### Population

The population of this descriptive and cross-sectional study consisted of 74 patients at a dialysis unit of a university hospital in Bursa, Türkiye. The endothelium is selectively permeable for macromolecules. This contributes to the maintenance of intravascular and extravascular fluid balance. Diabetes and hypertension cause endothelial dysfunction. Since diabetes and hypertension are the most common diseases leading to the development of chronic kidney disease, different mechanisms contribute to the process in other diseases, and to standardise the sample as much as possible, only patients with diabetes and/or hypertension were included in the study. Before the study, the number of patients diagnosed with diabetes and/or hypertension was determined by taking the list of patients registered at the haemodialysis centre (49 patients). The sample number was determined based on these patients. Individuals with chronic diseases other than diabetes and hypertension were excluded from the study because their diseases (18–65 years, rheumatoid arthritis, hepatitis, cancer) and the treatments they received could affect the level of fatigue (25 patients). A simple random sampling method was used in the study. The sample size was determined as 42 with a 90% confidence interval, with the sample size calculation knowing the population. Of the 49 patients who met the inclusion criteria, 42 patients aged 18–65 years who agreed to participate in the study constituted the study sample.

### Data Collection

A 20-question “General Information Form” (sociodemographic characteristics, health status), “Fatigue Severity Scale,” and “Dialysis Diet and Fluid Non-adherence Questionnaire” were prepared by the researchers in line with the literature. These forms were administered by face-to-face interview method before the haemodialysis procedure. Approximately 30 minutes of interview time was allocated for each patient.

In dialysis patients, fluid intake is less/more than necessary, and the patient’s non-compliance with the treatment and/or diet causes fluid imbalance. As a result, the patient’s weight increases or decreases. It was thought that this might cause temporary changes in the patient’s weight and may not clearly show the nutritional status but may be a sign of fluid and dietary restriction. The body mass index of the patients was calculated with the formula kg/m^2^ using the weight values measured before haemodialysis treatment in the haemodialysis unit and the results of the height measured during the registration of the patient to the haemodialysis unit. The investigators measured blood pressure values before the dialysis session. Individuals with blood pressure values above 130/80 mmHg were considered hypertensive, and those with blood pressure values below 90/60 mmHg were considered hypotensive. Laboratory values were used in the routine monitoring of patients in the haemodialysis unit. The last laboratory values in the patient files were taken before the dialysis session.

### Fatigue Severity Scale (Fss)

The Turkish validity and reliability of the scale developed by Krupp in 1989 was performed by Armutlu et al.^(8)^. FSS is a one-dimensional scale. Participants rate each item on a scale from 1 to 7, where a score of 1 indicates that they completely disagree with the statement in the relevant item, and a score of 7 indicates that they completely agree with it. The scoring of the scale consisting of a total of 9 questions varies between 9 and 63. A score of 36 or higher indicates severe fatigue. The total score is calculated by averaging the scores of 9 items. The cut-off value for pathological fatigue is determined as 4 and above. The lower the total score, the less the fatigue^(8)^. Cronbach’s alpha reliability coefficient of the FSS is 0.96. In this study, the Cronbach’s alpha reliability coefficient of the scale was determined to be 0.96.

### The Dialysis Diet and Fluid Non-Adherence Questionnaire (Ddfq)

The DDFQ was developed by Vlaminck et al.^(9)^, and its Turkish validity and reliability were performed by Kara^(10)^. The DDFQ, which evaluates nonadherence to diet and fluid intake restrictions in HD patients, is a self-report tool consisting of four subscales. The first and second statements in the scale are related to diet, and the third and fourth statements are related to non-compliance behaviour in terms of frequency and degree. The frequency of non-compliance with diet and fluid restriction is assessed by the number of days in the last 14 days when non-compliance behaviour was observed. The degree of non-compliance with diet and fluid restriction is scored between 0 and 4 (No non-compliance = 0, Mild = 1, Moderate = 2, Severe = 3, Very severe = 4).

### Data Analysis

The data were evaluated in the SPSS 22.0 program using percentages, averages, one-way ANOVA, T-tests, and Pearson correlation tests.

### Ethical Approval and Consent to Participate

Prior to the study, permission was obtained from the institution and the Ethics Committee (Uludağ University Faculty of Medicine Clinical Research Ethics Committee/2018-11/12). Throughout the study, the Declaration of Helsinki was adhered to, and participation in the survey was voluntary.

## RESULTS

The mean age of the subjects in the study was 51.78 ± 16.64 years, and 47.6% were female. 59.5% of the subjects lived in the city centre, and 35.7% came from the Marmara Region. Most (64.3%) were married, and 23.8% had children. Most were unemployed; 31% were primary school graduates, and 38.1% were retired. Moreover, 73.8% of the cases defined the economic situation of their families as income-expenditure equality. In 9.5% of the cases, there was a family member with renal failure. When the habits of the subjects were questioned, it was determined that 7.1% were smokers, 2.4% used alcohol, and 40.5% exercised regularly. The mean blood pressure of them was 51.78 ± 16.64, and 31% were hypertensive. The mean body mass index was 26.27 ± 4.80; 19% were obese, and 40.5% were overweight. Other chronic diseases were present in 40.5%, and hypertension in 23.8%. Of the subjects with another chronic disease (40.5%), 23.8% had hypertension, and 52.4% had undergone surgery (hernia surgery 14.3%). The mean duration of dialysis was 6.27 ± 5.83 years, and the mean number of weekly dialysis sessions was 2.85 ± 0.52. The mean weight of the patients at the time of diagnosis was 74.47 ± 20.99, the mean weight difference between two dialysis sessions was 1.89 ± 0.97, and 97.6% of the patients regularly visited the doctor. Most of the patients (83.3%) stated that they followed the diet recommended by their physician. In the laboratory findings of the cases, mean haemoglobin, haematocrit, and erythrocyte values were low; HbA1c, urea, creatinine, and ferritin values were high. The mean fatigue severity score was 24.78 ± 14.04, and 88.1% of the patients presented with fatigue. The mean number of days in the last two weeks when the patients did not follow their diets was 3.69 ± 4.85, and 2.4% of the patients did not follow it at all. The mean number of days in the last two weeks when the patients did not comply with fluid restriction was found to be 2.71 ± 5.02, and 2.4% did not comply with it at all. It was found that there was a significant relationship and difference between the sociodemographic characteristics, such as age and marital status, and severity of fatigue (p < 0.05) (Table 1).

**Table 1:** The effect of sociodemographic characteristics and health-related features on fatigue severity (N = 42) – Bursa, Turkey, 2018.

Variable	Mean ± SS		Significance	Variable	Mean ± SS		Significance
Age			^¥¥¥^r = 0,367*	Regular exercise status	Yes	21,70 ± 13,35	t = –1,177
			**p = 0,017**		No	26,88 ± 14,37	p = 0,246
Sex	Female	28,80 ± 13,62	^¥¥^t = 1,815	Blood pressure (systolic)			r = 0,033
	Male	21,13 ± 13,70	P = 0,077				p = 0,834
The place where the patient’s spent most of the life	City Center	27,04 ± 14,25	^¥^F = 0,793	Blood pressure (diastolic)			t = 0, 013
	County	21,25 ± 12,33	p = 0,460				p = 0,933
	Village	22,00 ± 17,44				
Marital status	Married	28,59 ± 11,95	^¥¥^t = 2,504	Blood pressure	hypotensive	29,27 ± 11,99	F = 1,304
	Single	17,93 ± 15,29	**p = 0,016**		normal	25,38 ± 15,13	p = 0,283
					hypertensive	20,15 ± 13,67	
Educational status	Illiterate	33,33 ± 7,81	^¥^F = 1,747	Degree of obesity	Weak	20,50 ± 28,99	F = 3,461
	Literate	16,00 ± 22,62	p = 0,149		Normal	17,93 ± 13,49	**p = 0,026** *
	Primary School	30,23 ± 11,76			Overweight	26,17 ± 13,17	
	Secondary School	21,75 ± 16,14			Obese	35,75 ± 5,14	
	High School	19,66 ± 15,25						
	University	16,25 ± 10,71					
Profession	Retired	25,05 ± 14,27	^¥^F = 1,447	Body Mass Index			r = 0,445
	Worker	23,00 ± 14,00	P = 0,244				p = 0,003*
	Housewife	29,42 ± 11,50					
	Other	16,75 ± 16,42					
Working status	No	25,57 ± 14,28	^¥¥^t = 1,132	Presence of obesity	Yes	22,20 ± 14,26	t = –2,624
	Yes	17,25 ± 9,74	p = 0,264		No	35,75 ± 5,14	**p = 0,012** *
Economic Condition of the family	Income < Expenses	28,44 ± 14,00	^¥^F = 1,445	The presence of an individual with chronic kidney failure in the family	Yes	29,25 ± 14,17	t = 0,664
	Income=Expenses	24,67 ± 14,08	p = 0,248		No	24,31 ± 14,13	p = 0,511
	Income > Expenses	10,00 ± 2,828					
Smoking Status	Smoker	31,66 ± 2,30	F = 0,392	History of any an surgical operation	No	23,86 ± 13,85	t = –0,442
	Quit	24,90 ± 13,71	p = 0,678		Yes	25,80 ± 14,54	p = 0,661
	Non-Smoker	24,00 ± 14,95					
Alcohol use	Drinker	29,00 ± 0,00	F = 0,052	Another chronic disease	No	30,00 ± 15,57	t = 0,566
	Quit	25,66 ± 11,01	p = 0,950		Yes	25,00 ± 14,69	p = 0,582
	Non-Drinker	24,60 ± 14,54					

* p < 0.05 F = One-way Anova test, t = t test, r = Pearson correlation test.

The sociodemographic characteristics of the subjects did not affect the number of days of dietary non-compliance in the last two weeks (p > 0.05). Also, the health characteristics of the subjects did not show significant relationships or differences (p > 0.05) with the number of days of non-adherence to diet in the last two weeks (Table 2).

**Table 2:** The effect of socio-demographic characteristics and health-related features on the number of days in the last two weeks when the patients did not adhere to their diet (N = 42) – Bursa, Turkey, 2018.

Variable	Mean ± SS		Significance	Variable	Mean ± SS		Significance
Age			r = 0,098	Regular exercise status	Yes	2,94 ± 4,00	t = –0,821
			p = 0,537		No	4,20 ± 5,37	p = 0,416
Sex	Female	3,65 ± 4,94	t = –0,051	Blood pressure (systolic)			r = 0,055
	Male	3,72 ± 4,89	p = 0,960				p = 0,727
The place where the patient’s spent most of the life	City Center	3,92 ± 4,64	F = 0,075	Blood pressure (diastolic)			t = –0, 044
	County	3,25 ± 5,20	p = 0,928				p = 0, 780
	Village	3,60 ± 6,06					
Marital status	Married	3,07 ± 4,37	t = –1,107	Blood pressure	hypotensive	2,90 ± 4,78	F = 0,201
	Single	4,80 ± 5,60	p = 0,275		normal	3,83 ± 5,14	p = 0,819
					hypertensive	4,15 ± 4,81	
Educational status	Illiterate	1,83 ± 3,25	F = –1,954	Degree of obesity	Weak	4,00 ± 5,65	F = 0,674
	Literate	1,00 ± 1,41	p = 0,099		Normal	1,80 ± 2,67	p = 0,305
	Primary School	3,61 ± 4,89			Overweight	4,58 ± 5,72	
	Secondary School	1,25 ± 2,05			Obese	5,25 ± 5,65	
	High School	7,55 ± 5,57					
	University	5,66 ± 7,23					
Profession	Retired	4,06 ± 5,27	F = –0,275	Body Mass Index			
	Worker	7,50 ± 9,19	p = 0,785			3,32 ± 4,66	t = –1,010
	Housewife	3,28 ± 4,85				5,25 ± 5,65	p = 0,319
	Other	3,00 ± 3,89					
Working status	No	3,50 ± 4,78	t = –0,731	Presence of obesity	Yes		r = 0,296
	Yes	5,66 ± 7,23	p = 0,469		No		p = 0,057
Economic Condition of the family	Income < Expenses	5,33 ± 5,59	F = 0,995	The presence of an individual with chronic kidney failure in the family	Yes	3,18 ± 4,77	t = –0,708
	Income = Expenses	3,41 ± 4,72	p = 0,379		No	4,25 ± 5,00	p = 0,483
	Income > Expenses	0,50 ± 0,70					
Smoking Status	Smoker	1,66 ± 2,88	F = 0,636	History of any an surgical operation	No	4,25 ± 6,65	t = 0,239
	Quit	4,90 ± 6,00	p = 0,535		Yes	3,63 ± 4,74	p = 0,812
	Non-Smoker	3,42 ± 4,54					
Alcohol use	Drinker	5,00 ± 0,00	F = 0,510	Another chronic disease	No	3,47 ± 4,83	t = –0,239
	Quit	1,00 ± 1,00	p = 0,605		Yes	3,84 ± 4,96	p = 0,812
	Non-Drinker	3,86 ± 5,04					

*p < 0.05 F = One-way Anova test, t = t test, r = Pearson correlation test.

The sociodemographic characteristics of the subjects did not affect the number of days of non-compliance with fluid restriction (p > 0.05). However, the presence of chronic disease led to a significant difference in the number of days of non-compliance with fluid restriction in the last two weeks (p < 0.05) (Table 3).

**Table 3:** The effect of sociodemographic characteristics and health-related features on the number of days in the last two weeks when the patients did not adhere to fluid restriction (N = 42) – Bursa, Turkey, 2018.

Variable	Mean ± SS		Significance	Variable	Mean ± SS		Significance
Age			r = –0,207	Regular exercise status	Yes	2,70 ± 4,98	t = –0,009
			p = 0,188		No	2,72 ± 5,15	p = 0,993
Sex	Female	2,80 ± 5,36	t = 0,104	Blood pressure (systolic)			r = –0,121
	Male	2,63 ± 4,81	p = 0,918				p = 0,444
The place where the patient’s spent most of the life	City Center	3,04 ± 5,35	F = 0,824	Blood pressure (diastolic)			t = –0,132
	County	3,16 ± 5,27	p = 0,446				p = 0,405
	Village	1,00 ± 0,00					
Marital status	Married	2,55 ± 4,68	t = –0,272	Blood pressure	hypotensive	4,00 ± 5,79	F = 2,362
	Single	3,00 ± 5,74	p = 0,787		normal	0,83 ± 3,29	p = 0,108
					hypertensive	4,23 ± 5,79
Educational status	Illiterate	0,66 ± 1,63	F = 1,925	Degree of obesity	Weak	1,00 ± 0,00	F = 0,674
	Literate	1,50 ± 2,12	p = 0,118		Normal	2,53 ± 4,82	p = 0,573
	Primary School	3,30 ± 5,49			Overweight	2,23 ± 4,64	
	Secondary School	0,75 ± 1,75			Obese	4,75 ± 6,67	
	High School	6,44 ± 7,19					
	University	1,00 ± 0,00					
Profession	Retired	2,05 ± 4,58	F = –0,440	Body Mass Index			r = 0,244
	Worker	2,00 ± 2,64	p = 0,725				p = 0,119
	Housewife	4,00 ± 6,07					
	Other	2,12 ± 4,91					
Working status	No	2,86 ± 5,21	t = 0,608	Presence of obesity	Yes	2,23 ± 4,54	t = –1,284
	Yes	1,25 ± 2,50	p = 0,547		No	4,75 ± 6,67	p = 0,207
Economic Condition of the family	Income < Expenses	4,11 ± 5,92	F = 0,661	The presence of an individual with chronic kidney failure in the family	Yes	2,63 ± 4,81	t = –0,104
	Income = Expenses	2,48 ± 4,89	p = 0,522		No	2,80 ± 5,36	p = 0,918
	Income > Expenses	1,00 ± 0,00					
Smoking Status	Smoker	1,66 ± 2,88	F = 0,067	History of any an surgical operation	No	30,50 ± 7,00	t = 0,325
	Quit	2,81 ± 5,56	p = 0,935		Yes	2,63 ± 4,89	p = 0,747
	Non-Smoker	2,78 ± 5,10					
Alcohol use	Drinker	5,00 ± 0,00	F = 0,272	Another chronic disease	No	5,29 ± 6,33	t = 3,001
	Quit	1,00 ± 1,00	p = 0,763		Yes	0,96 ± 2,90	p = 0,005*
	Non-Drinker	2,78 ± 5,24					

*p < 0.05 F = One-way Anova test, t = t test, r = Pearson correlation test.

Laboratory values and creatinine and calcium levels were correlated with the number of days of non-compliance with fluid restriction in the last two weeks and the degree of non-compliance with fluid restriction (p < 0.05) (Table 4).

**Table 4:** The relationship between the laboratory values, the severity of fatigue, and the number of days in the last two weeks when the patients did not comply with their diet and fluid restriction (N = 42) – Bursa, Turkey, 2018.

		hb	hct	ert	albumin	t.kolesterol	hdl	ldl	aks	hba1c	üre	kreatinin	alt	ast	na	k	ca	wbc	ferritin
Fatigue	r	–,091	–,063	–,036	–,198	–,017	–,045	–,080	,079	,100	,079	,092	,053	,026	,108	–,124	,055	,199	,108
	p	,568	,692	,819	,209	,917	,776	,615	,620	,530	,620	,562	,738	,869	,498	,434	,728	,207	,496
The number of days that she has not followed the diet in the last two weeks	r	,006	,133	–,025	–,147	,001	–,118	,148	,065	,213	,097	,229	–,066	–,202	,047	,136	,004	,216	,061
	p	,972	,401	,873	,353	,993	,456	,351	,682	,175	,543	,144	,680	,199	,770	,391	,982	,170	,703
The number of days that did not comply with the fluid constraint in the last two weeks	r	,033	,162	,134	–,092	–,026	–,149	,089	–,145	,116	,037	,310*	–,111	–,234	,199	,043	–,429**	,073	–,074
	p	,835	,304	,397	,563	,872	,346	,574	,358	,464	,816	**0,046**	,485	,135	,206	,789	**0,005**	,644	,641
Degree of noncompliance with diet in the last two weeks	r	,165	,226	–,005	–,265	,145	–,090	,192	,105	,099	,251	,259	–,071	–,171	,139	,008	,002	,115	,191
	p	,297	,151	,973	,090	,360	,572	,224	,508	,533	,109	,098	,653	,279	,381	,961	,990	,470	,226
Degree of noncompliance with fluid restriction in the last two weeks	r	,040	,143	,162	–,024	,010	–,199	,007	–,159	,141	,205	,411**	–,122	–,187	,214	,154	–,424**	,075	–,016
	p	,802	,366	,305	,880	,950	,207	,967	,315	,372	,193	**,007**	,441	,236	,173	,329	**,005**	,635	,920

* p < 0.05 r = Pearson correlation test.

No significant correlation was found between the severity of fatigue and the number of days of non-compliance with diet and fluid restriction and the degree of non-compliance with diet and fluid restriction in the last two weeks (p > 0.05) (Table 5).

**Table 5:** The relationship between the severity of fatigue and the number of days in the last two weeks when the patients did not comply with their diet and fluid restriction (N = 42) – Bursa, Turkey, 2018.

		The number of days that she has not followed the diet in the last two weeks	The number of days that did not comply with the fluid constraint in the last two weeks	Degree of noncompliance with diet in the last two weeks	Degree of noncompliance with fluid restriction in the last two weeks
Total fatigue	r	0,147	0,229	0,034	0,034
	P	0,352	0,144	0,830	0,830

*p < 0.05 r = Pearson correlation test.

## DISCUSSION

### Fatigue Status of the Patients and Their Adherence to Diet and Fluid Restriction

Dialysis patients are typically one of the patient groups with the highest rate of non-compliance with treatment and treatment-related processes. While 6%–23% of dialysis patients do not comply with drug treatment, 7%–32% do not comply with dialysis treatment sessions, 31% interrupt dialysis treatment, and 18% do not even attend dialysis sessions^(11,12)^.

The present study found that the mean number of days in the last two weeks when the patients did not comply with their diets was significantly higher than the mean number of days when they did not comply with their diets. In previous studies, the frequency of non-compliance with dietary treatment in the last two weeks was found to vary between 3.42 ± 3.9 and 7.4 ± 6.1. In related studies, non-compliance with dietary therapy was found to vary between 14% and 98.3%^(11–13)^. The findings of this study are in parallel with the studies conducted on different patient groups receiving CRF and haemodialysis treatment.

This study observed that the average number of days during the last two weeks when patients did not comply with fluid restriction was quite high. In previous studies, the frequency of non-compliance with fluid restriction in the last two weeks was found to vary between 2.76 ± 3.98 and 6 ± 5.6^(14)^. In other studies, it was found that non-compliance with fluid restriction was in varying degrees, and the frequency ranged between 18% and 95%^(11–13)^. The results of this study are similar to the results of studies conducted in different patient groups receiving CRF and haemodialysis treatment.

Dialysis patients usually do not comply with their treatment, diet, or fluid restrictions. The reason for this is the physiological and metabolic changes resulting from dialysis application, as well as the psychological problems resulting from the effect of dialysis on the patient’s whole life and the inability to continue their work with their former competence^(15)^.

### The Effect of Sociodemographic Features on Fatigue

In this study, age, one of the sociodemographic characteristics, was found to be effective in the severity of fatigue. In studies conducted to date, a positive relationship between age and fatigue has been demonstrated, as well^(16,17)^. In a study conducted in Egypt, it was suggested that age did not affect the mean fatigue score, but the scores of those over 69 years of age were higher than those of other age groups^(18)^. Changes in the respiratory and cardiovascular system, decreased tissue perfusion, decreased renal function, and fluid-electrolyte imbalances caused by increased urea result in fatigue as the individual ages^(19)^. In addition, weight loss occurs with decreased adipose tissue due to nutritional problems as a result of gastrointestinal system changes and endocrine system changes in elderly patients, and interleukin level decreases with weight loss, which leads to fatigue in the elderly^(20)^. The results of this study are generally compatible with the literature.

This study found that the mean fatigue score of females was higher than that of males. In the studies conducted to date, it has been determined that the mean fatigue score is higher in females than in males^(21)^. The frequency of iron deficiency is very high in females in Türkiye. It is thought that the reason for the higher level of fatigue in females is culturally related to the responsibilities imposed on females by housework and child-rearing. In this study, it was found that the marital status of the patients was influential on the severity of fatigue, and the mean fatigue scores of married patients were higher than those of single patients. Married people defined physical fatigue as significant changes in weight, chronic back pain, stomachache, headache, and chronic insomnia; mental fatigue as a state of negativity in their relationships with others and self-concept; and emotional fatigue as hopelessness and disappointment. The responsibilities of individuals increase with marriage. There is a relationship between the duration of marriage and marital status and the levels of mental, emotional, and physical fatigue in marriage^(22)^. It is thought that the higher level of fatigue observed in married individuals in our study is due to increased stress with anxiety, livelihood responsibilities, and accompanying economic problems.

In this study, it was found that illiteracy did not statistically affect the severity of fatigue. However, the fatigue severity of illiterate patients was found to be higher than that of other patients. A study on fatigue and quality of life in patients with cancer found that Piper Fatigue Scale scores decreased with increasing education levels^(23)^. In cancer patients, fatigue is observed more frequently and more severely since the treatment process is long, and factors such as deterioration of physical and psychological health accompany the process in addition to anaemia^(24)^. It is thought that as the knowledge and awareness levels of individuals in other subjects increase, their knowledge and awareness about health increase accordingly, and as their knowledge and awareness about health protection and development increase, they can cope with the symptoms of their diseases, such as fatigue, more easily.

### The Effect of Health-Related Features on Fatigue

In this study, it was determined that body mass index and the presence and degree of obesity affected the severity of fatigue. In various studies conducted with different patient groups, it was found that there was a relationship between obesity/body mass index and fatigue, and fatigue levels were higher in obese individuals. However, there is a negative relationship between obesity/body mass index and fatigue. There are also studies showing that there is no relationship between body mass index and fatigue^(25,26)^.

Fatigue in individuals is a factor that prevents physical activity. As a result of the inability to perform physical activity, the frequency of obesity increases in patients. While the results of this study are similar to those of some other studies, they differ from those of others. Increasing body mass index causes a decrease in physical activity and increases oxygen consumption and carbon dioxide production in the body. However, fatigue may also occur in individuals due to changes in the respiratory, circulatory, and excretory systems due to chronic diseases and related complications, as well as effects observed in haematopoiesis and metabolic processes^(27)^.

### Examination of the Effects of Sociodemographic Features on Adherence to Diet and Fluid Restriction

The present study found that sociodemographic characteristics did not affect the number of days in the last two weeks when patients did not comply with their diets. In a study conducted by Acar in CRF patients, it was found that gender affected compliance with diet and fluid restriction, and males showed less compliance with diet than females^(23)^. Another study on haemodialysis patients found a significant difference between the duration and severity of dietary non-compliance and marital status^(14)^. In this study, the results differ from the literature. The reason for this is thought to be the sizes of the samples included in the studies, the marital status of the samples, and the differences in the mean age.

In this study, it was determined that the sociodemographic characteristics of the patients did not affect the number of days when they did not comply with fluid restriction. In some of the studies conducted to date, it was found that sociodemographic characteristics did not affect compliance with fluid restriction^(13)^. In contrast, in some studies, childbearing and educational status among sociodemographic characteristics were found to affect compliance with fluid restriction^(28)^. The difference in study results is thought to be due to sample size, inclusion criteria, and the socio-cultural characteristics of the city/country where the studies were conducted.

### Effect of Health Status on Adherence to Diet and Fluid Restriction

The present study determined that the presence of another chronic disease affected the number of days in the last two weeks when the subjects did not comply with fluid restriction. In studies conducted on haemodialysis patients, no significant difference was found between the frequency of non-compliance with diet and fluid restriction and the presence of comorbid disease^(13)^. Hence, the results of this study are not in parallel with the literature. This is thought to be due to the comorbid conditions in the sample groups and the differences in the duration and frequency of dialysis.

This study found a correlation between the creatinine and calcium values of the patients and the number of days in the last two weeks when they did not comply with fluid restriction. Studies have found that non-compliance affects laboratory findings such as phosphorus value^(10,13)^. In terms of the effect of non-compliance with fluid restriction on laboratory values, the results of this study are similar to the literature. However, they differ in terms of the affected mineral value. The reason for this is thought to be that the stage of renal failure was different in the sample groups, and laboratory values were affected by many factors, such as having another chronic disease and duration and frequency of dialysis. In addition, it is thought that the dilution of electrolytes, blood cells, and waste products in plasma with increasing fluid volume affects the laboratory results.

### Examining the Relationship Between Fatigue Severity and Adherence to Diet and Fluid Restriction

In this study, there was no significant correlation between fatigue severity, the number of days in the last two weeks when the patients did not comply with dietary and fluid restrictions, and the degree of non-compliance with dietary and fluid restrictions. In a study comparing the quality of life of patients with chronic renal failure who had not yet started dialysis treatment and patients receiving haemodialysis treatment, no relationship was found between diet, compliance with treatment, and fatigue^(29)^. In another study on patients receiving haemodialysis treatment, no significant relationship was found between compliance with special diet programmes and general fatigue levels^(30)^. The results of this study are similar to the findings of other studies.

### Limitations of the Study

The results of the study cannot be generalised to all haemodialysis patients because the study was conducted in a single centre, and the sample size was small. In addition, although the patients met the inclusion criteria at the beginning of the study, they were excluded from the sample due to complications that developed during the study, which constitutes the limitation of the study.

## CONCLUSION

In haemodialysis patients, the inability to adjust fluid-electrolyte balance and changes in haematopoietic, metabolic, and endocrine functions cause individuals to feel severe fatigue. Biological and psychological problems may occur due to fatigue, and social relations and work life are negatively affected. Although no relationship was found between compliance with diet and fluid restriction and the severity of fatigue in this study, it is thought that other factors affecting fatigue in dialysis patients may have affected this result. It is recommended to conduct randomised controlled studies in which the factors affecting patient fatigue are controlled. It is thought that ensuring weight control in patients will effectively reduce fatigue. One of the factors affecting patient compliance with dietary recommendations and fluid restriction is the communication between the patient, physician, and nurse. It is recommended that a counselling service should be structured to facilitate the patient’s communication with the team providing treatment and care. To ensure compliance with treatment and diet, comprehensive (such as diet, fluid intake, and factors affecting fatigue) and standardised training should be given to patients by nurses, and continuous patient follow-up should be performed by nurses. In the centre where the study was conducted, nurses provided adequate patient training, and patient follow-up was performed effectively. As a result, it is thought that patients’ compliance with treatment and diet increased. It is recommended that the study be replicated with a larger sample to generalise the results to haemodialysis patients.
